# RETRACTED: Tian et al. The cGAS-STING Pathway: A New Therapeutic Target for Ischemia–Reperfusion Injury in Acute Myocardial Infarction? *Biomedicines* 2024, *12*, 1728

**DOI:** 10.3390/biomedicines13112818

**Published:** 2025-11-19

**Authors:** Mengxiang Tian, Fengyuan Li, Haiping Pei

**Affiliations:** 1 Department of General Surgery, Xiangya Hospital, Central South University, Changsha 410083, China; 2 National Clinical Research Center for Geriatric Disorders, Xiangya Hospital, Central South University, Changsha 410083, China

The Journal retracts the article “The cGAS-STING Pathway: A New Therapeutic Target for Ischemia–Reperfusion Injury in Acute Myocardial Infarction?” [1] cited above.

Following publication, the authors contacted the Editorial Office with concerns regarding the use of images without appropriate permission and the originality of the conclusions presented in this review article.

Adhering to our standard procedure, an investigation was conducted by the Editorial Office and Editorial Board which confirmed a lack of appropriate permission to publish material presented in this article. Further concerns relating to the appropriateness of the authorship list, the accuracy of the contribution section, and the origins of the study could not be fully resolved. Consequently, the Editorial Board has lost confidence in the reliability of the findings and has decided to retract this publication, as per MDPI’s retraction policy (https://www.mdpi.com/ethics#_bookmark30).

This retraction was approved by the Editor-in-Chief of the *Biomedicines*.

The authors did not provide a comment on this decision.
